# Effects of a Person-Centered Nursing Intervention for Frailty among Prefrail Community-Dwelling Older Adults

**DOI:** 10.3390/ijerph17186660

**Published:** 2020-09-13

**Authors:** Jiyeon Ha, Yeon-Hwan Park

**Affiliations:** 1 College of Nursing, Konyang University, Daejeon 35365, Korea; jyhaha403@gmail.com; 2 College of Nursing, Seoul National University, Seoul 03080, Korea; 3 The Research Institute of Nursing Science, Seoul National University, Seoul 03080, Korea

**Keywords:** frailty, aged, person-centered therapy, primary prevention, senior centers

## Abstract

This study examined the effects of a person-centered nursing intervention program for frailty (PNIF) targeting community-dwelling prefrail older people in South Korea. The study participants were 40 community-dwelling older adults (≥65 years) who were classified as prefrail on the Cardiovascular Health Study (CHS) frailty index. The intervention group (*n* = 20) received group intervention sessions two days/week for twelve weeks and the control group (*n* = 20) attended lectures about frailty prevention one day/week for four weeks. The evaluation instruments included the CHS Frailty Index, a JAMAR^®^ hydraulic hand dynamometer, the Short Physical Performance Battery (SPPB), the Korean version of the Community Healthy Activities Model Program for Seniors Questionnaire (K-CHAMPS), the Mini Nutritional Assessment (MNA), the Geriatric Depression Scale Short Form-Korea Version (GDSSF-K), the ENRICHD Social Support Instrument (ESSI), and the Goal Attainment Scale (GAS). Significant differences were found in the CHS Frailty Index (*p* < 0.001), left-hand grip strength (*p* = 0.022), right-hand grip strength (*p* = 0.009), SPPB (*p* = 0.007), K-CHAMPS (*p* = 0.009), MNA (*p* = 0.018), and GDSSF-K (*p* = 0.001) between the two groups after 12 weeks. No significant between-group differences in ESSI scores were observed. The PNIF effectively improved grip strength, physical function, physical activity, and nutritional status, reduced depression, and prevented frailty among community-dwelling older adults.

## 1. Introduction

South Korea has one of the fastest growing aging populations in the world. The rise in the elderly population worldwide has also led to an increase in the prevalence of chronic conditions and comorbidities [1]. The prevalence of chronic conditions and multiple chronic conditions among older adults (≥65 years) in South Korea has been reported to be 90.4% and 72.2%, respectively [2]. Older people with multiple chronic conditions are at an increased risk of frailty, which increases dependence due to physical and mental impairment [3]. Frailty in older adults is defined as a state of diminished overall ability to maintain the ability to live independently, and frail older adults appear vulnerable and lack vigor [4]. A systematic review that analyzed 21 frailty-related studies reported that the prevalence of frailty and prefrailty in community-dwelling older adults was approximately 10.7% and 41.9%, respectively [5]; in South Korea, the rates of frailty and prefrailty among older adults (aged 65 years or older) were reported to be 11.7% and 38.8%, respectively [6].

Frailty is typically a result of factors such as reduced muscle strength and physical function, lack of physical activity, chronic undernutrition, depression, and decreased social support [7]. The accumulation of these factors induces frailty, and if left untreated, it is likely to progress to an irreversible disability, detrimental to older adults’ independent functioning [8]. Frailty not only diminishes older people’s quality of life by increasing the incidence of falls, cardiovascular diseases, hypertension, and cancer, as well as hospital and nursing home admissions, but also may ultimately lead to fatal outcomes [3,9]. Moreover, the loss of independence and the need for long-term care are among the major contributors to elevated national healthcare costs, placing a grave burden on individuals, families, and nations [8].

Individuals undergo dynamic transitions between levels of frailty, such as non-frailty, prefrailty, and frailty. Interventions to prevent frailty should be designed to be more effective by targeting specific levels of frailty [10]. The prefrail state is the intermediate stage between non-frailty and frailty. In this state, individuals have a significantly higher risk than non-frail older adults to progress to negative health outcomes but have the potential to recover to a healthy state through physiologically appropriate responses to acute illness and stress [9]. Once an older adult becomes prefrail, however, they are more likely to progress to frailty. Therefore, identifying those who are prefrail and delivering appropriate interventions are useful and cost-effective measures to prevent or delay frailty [8,11].

Although many previous studies have recognized the importance of frailty in older adults and have developed some frailty interventions with positive outcomes, frailty-related research is still at an inchoate stage [12]. Studies focusing on preventive interventions for prefrail older adults are especially lacking [11]. While modalities such as exercise, nutrition, technological interventions, and hormone replacement therapies have been proposed as components of frailty prevention programs, most studies have focused on exercise-based interventions for physical frailty [13]. However, the recent recognition of the multidimensional properties of frailty has highlighted the need for individualized multifactorial interventions, such as those targeting the physical and psychosocial domains of health [8]. However, most of these studies focused on low-income older adults registered with home-based health management projects or residents of long-term care facilities. They have rarely examined the effectiveness of interventions administered to community-dwelling prefrail older adults, who are not candidates for these services but are at risk of frailty [4,14]. Furthermore, even some studies that took into consideration the multidimensional nature of frailty provided only lecture-based interventions, thereby failing to consider older adults’ individual characteristics, preferences, and environments [14,15].

Beswick and colleagues [16] stated that frailty interventions failed to improve physical function and quality of life because the specific needs of individuals were not evaluated prior to implementing the intervention. Person-centered interventions are designed to reflect personal values, preferences, and needs based on the philosophy of person-centeredness, so they may be useful as nursing interventions, as they are suitable to the traditional nursing philosophy that values individualized care [17,18,19]. As older adults have complex nursing needs that impact their daily living [20], and even those of the same age vastly differ in their health status depending on factors such as degree of aging, current illness, and physical functions [21], individualized, person-centered interventions that consider individuals’ functional levels and features are warranted. In the present study, we devised and implemented an intervention for older adults that reflected individual preferences through goal-setting and monitoring using the Goal Attainment Scale (GAS), a face-to-face individual baseline survey, and regular telephone support. In particular, the participants of our intervention program were able to set their own goals for exercise and nutrition every week and make positive lifestyle changes as a result.

Therefore, this study aimed to examine the effects of a person-centered frailty prevention program for frailty (PNIF) that reflected the multidimensional properties of frailty, including muscle strength and physical function, physical activities, nutrition, depression, and social support, taking into consideration individuals’ health status, functional level, and preferences. We hypothesized that, compared with participants assigned to the control group, the participants in the PNIF would show significant improvement in (1) the Cardiovascular Health Study (CHS) Frailty Index, (2) grip strength, (3) the Short Physical Performance Battery (SPPB), (4) the Korean version of the Community Healthy Activities Model Program for Seniors (K-CHAMPS), (5) Mini Nutritional Assessment (MNA), (6) the Geriatric Depression Scale Short Form-Korean Version (GDSSF-K), and (7) the ENRICHD Social Support Instrument (ESSI).

## 2. Materials and Methods

### 2.1. Study Design

This study was a quasi-experimental pretest–posttest design applying a PNIF to community-dwelling prefrail older adults for 12 weeks.

### 2.2. Participants

The study participants were identified through convenience sampling from community-dwelling adults aged 65 years or older in the prefrailty stage who were registered with a senior center and expressed willingness to participate in the study. The specific inclusion criterion included persons classified as “prefrail” according to the CHS Frailty Index. The exclusion criteria were serious cognitive impairment (i.e., a Mini-Mental State Examination in the Korean version of the Consortium to Establish a Registry for Alzheimer’s Disease (CERAD) assessment packet (MMSE-KC) score > 18) and current participation or any plan to participate in another health-promotion program or similar program during the study period.

The mean age of the participants was 77.10 years for the intervention group and 79.30 years for the control group, and the percentage of women was 80.0% (*n* = 16) and 60.0% (*n* = 12) in the two groups, respectively. All 20 (100.0%) participants in the intervention group graduated from elementary school or higher, while two (10.0%) participants in the control group responded that they had received no formal education.

The sample size necessary to achieve the goals of this study was determined through statistical power analysis using G*Power version 3 [22]. Assuming a two-tailed test with a significance (α) of 0.05, power (1-β) of 0.8, and effect size (*d*) of 1.12 based on a prior study [12] that was similar to our study, the sample size required for each group was determined to be 14. Considering a 20–40% withdrawal rate during the intervention, at least 20 participants were recruited for both the intervention and control groups.

We administered the MMSE-KC and CHS Frailty Index at a senior center over two days to promote the program and perform screening, and a total of 57 participants were selected per the inclusion criteria. Thirteen individuals refused to participate in the study, and 44 provided written informed consent to participate in the study.

One data collector assigned each participant an identification number in the order in which they signed the consent form. A free web-based allocation program offered by the Social Psychology Network for researchers and students was used to assign 22 participants to the intervention group and 22 to the control group. In addition, in consideration of the diffusion effect, which could threaten the internal validity of the study, the timing of the program intervention group was different than that for the control group. To minimize participant contact, the dates for the baseline and post-intervention surveys were different and the locations for the intervention and data collection were separate.

**Figure 1:**
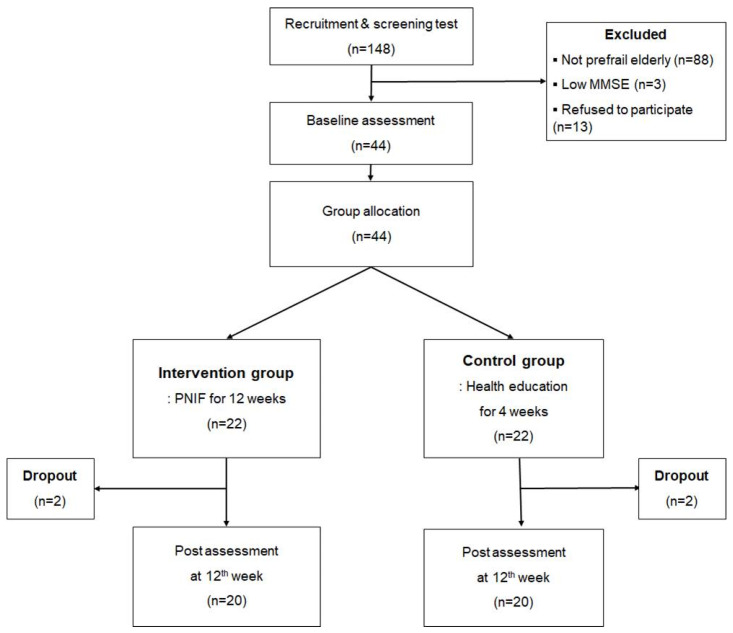
Flow diagram of this study.

After 12 weeks of the 24-session program, two participants from each group withdrew of their own accord; thus, 20 of the 22 participants in the intervention group and 20 of 22 participants in the control group completed both the baseline and post-intervention survey (Figure 1).

### 2.3. Ethical Considerations

This study was approved by the Institutional Review Board at Seoul National University (IRB No.1701/002-004) prior to data collection. The participants were given detailed oral and written explanations of the study’s purpose and procedures, the content of the program, and their ability to withdraw from the study at any point. All participants signed a written consent form prior to data collection and intervention. The participants were also informed that the collected data would be accessible only by the researchers and that all personal information would be kept confidential. We also specified that the collected data would be coded anonymously, would not be used for non-research purposes, and would be shredded and discarded or incinerated upon completion of the study. Both the intervention and control groups were given a gift after data collection as a token of appreciation for their participation.

### 2.4. Intervention Program

**Figure 2:**
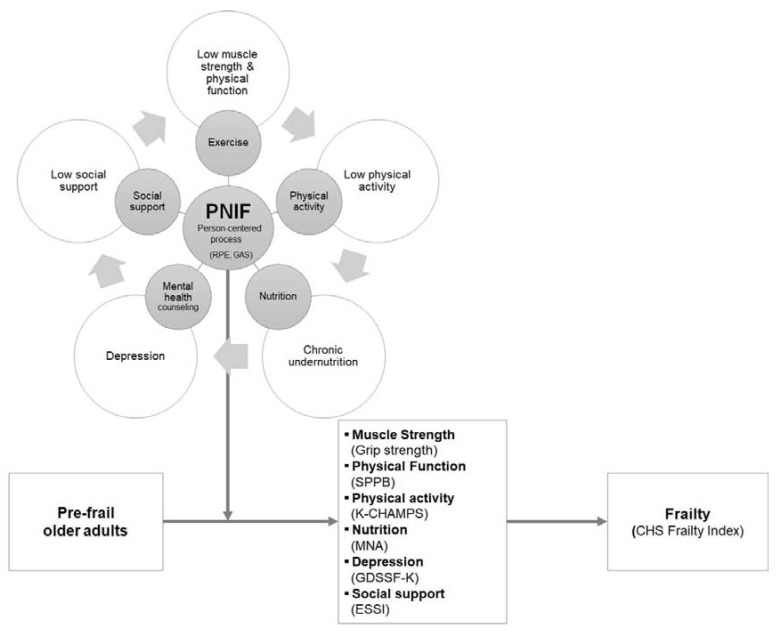
Conceptual framework of this study.

We developed a 24-session PNIF to be administered twice per week for 12 weeks for community-dwelling prefrail older adults, where exercise and physical activity (Session 1) and nutritional and psychosocial interventions (Session 2) were given in consideration of individuals’ level of health, preference, and needs. We used the cycle of frailty model presented by Fried and colleagues [23] as the theoretical framework but included psychosocial factors such as depression and social support to develop a multidimensional frailty prevention program (Figure 2). As the elderly population has a high prevalence of multiple chronic conditions and complex health status and needs, the causes of frailty vary widely across individuals; therefore, it is necessary to develop a program that encompasses various relevant factors to meet the diverse needs of community-dwelling older adults and bring about frailty prevention and improvement.

**Figure 3:**
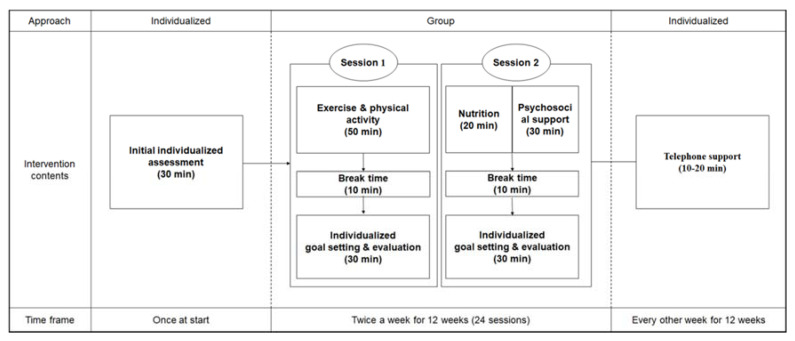
Program construct of the person-centered nursing intervention program for frailty (PNIF).

The intervention consisted of a 30-min initial individual evaluation, two sessions per week (90 min each), and 10–20 min of over-the-phone support. Each session consisted of group intervention and individual goal-setting and monitoring (Figure 3). All interventions were provided by the researcher with the support of two research assistants who were nursing students.

The individual goal-setting and monitoring and telephone support that followed the initial individual evaluation and group intervention were the primary focus of this program, as person-centered nursing strategies were implemented during these processes. These strategies enabled the identification of information regarding participants’ current disease and health status, needs or preferences, lifestyle, exercise, diet, psychological state, and level of social support. It also improved our understanding of the participants, which eventually enabled specific and realistic goal-setting and achievement. Six rounds of telephone support were provided, for 10–20 min per session, once every two weeks.

#### 2.4.1. Content of the PNIF

##### Exercise and Physical Activity Intervention (Session 1)

The muscle training regimen in Exercise for Frail Elders (second edition) by Martini and Jones-DiGenova [24] was used as the framework for increasing muscle strength and physical activities. Exercise for Frail Elders guides users with specific and practical methods and instructions for designing and providing successful exercise programs for older adults who were frail.

The exercises comprised 2–6 types of upper and lower limb resistance exercises, depending on the difficulty of each session, and the routine was repeated for 30 min under the direction of the researcher. Range of motion exercise was performed as a cool-down exercise. During the intervention, the Rate of Perceived Exertion (RPE) was measured. The intensities were adjusted to 9–16 RPE depending on the health status and feedback on the day of the session.

After exercise, we supported the participants to set new exercise goals for the week based on the Goal Attainment Scale (GAS) in the previous week and the exercise learned that day. If the participants had unrealistic expectations or anticipated overly negative outcomes, we assisted them in adjusting their goals appropriately. Typically, the baseline state refers to the current state of participant, and this state is referred to as “-1,” and the worst baseline state is “-2” [25]. It is important to guide participants to set specific, measurable, and realistic short-term goals that are achievable within the designated period [25]. We encouraged the participants to check their daily goal achievements using their daily self-monitoring journal at home.

##### Nutritional and Psychosocial Interventions (Session 2)

The nutritional intervention consisted of nutrition education and counseling tailored to individuals’ health status, chronic conditions, diet, household structure, and living environment. The intervention was administered once per week for approximately 20 min per session, and baseline nutritional assessment was performed to examine the individual’s diet and dietary preferences, level of independence from chronic disease, and allergies in detail. The Dietary Reference Intake values for Koreans were used as a reference for dietary intake. After administering nutritional education, participants were encouraged to set their own weekly goals to improve their diet, and the anticipated results were examined using the GAS. The GAS was used according to the same guidelines that were used for the exercise and physical activity interventions. Moreover, when evaluating the level of attainment of a previous goal, participants were instructed to write down the barriers to their goal attainment.

As a psychosocial intervention, group education and counseling regarding ways to protect mental health, such as depression relief and stress management, were provided. The group psychosocial intervention was conducted once per week for about 30 min per session. The content of Session 2 for each week is shown in Table A1.

#### 2.4.2. Control Group

The control group was given a lecture-based education on frailty prevention and management once per week for four weeks by the researcher. Each session lasted approximately 60 min and took place at a senior center. Session 1 covered the definition and current state of frailty among older adults, components of frailty, and the importance of self-care. Session 2 dealt with moderate- to vigorous-intensity muscle training for frailty prevention and the importance of increasing physical activity. Session 3 addressed the nutritional features and importance of nutritional management and dietary guidelines for older adults. Session 4 focused on the management of depression and mental health and the importance of social support.

### 2.5. Outcome Measurements

The variables and outcome measurement tools of this study are as follows Table A2.

#### 2.5.1. Demographic and Clinical Characteristics

The general characteristics analyzed herein included factors that have been reported to be associated with frailty in previous research, specifically age, sex, education level, marital status, household structure, religion, perceived economic status, and MMSE-KC score [15,23,26,27].

#### 2.5.2. Cardiovascular Health Study Frailty Index

This instrument was developed by Fried and colleagues [23], and five domains were used by Hwang and colleagues [6] For the Reduced Physical Activity Domain, however, we used the K-CHAMPS instead of the International Physical Activity Questionnaire that was used in a previous study [6]. Individuals were divided by sex, and a score of 1 was given to those in the bottom 20% of physical activity. For the Weight Loss Domain, participants were asked whether they had experienced a reduction in body weight of more than 4.5 kg or 5% in the past year, and a score of 1 was given to those who answered “yes.” For the Reduced Vitality Domain, a score of 1 was given to those who “felt exhausted with everything” or “felt that [they] cannot carry on anymore” for at least 3 days per week. For the Reduced Walking Speed Domain, 4.5-m walk speed was measured and adjusted by height and sex, and a score of 1 was given to the lowest 20%. For the Reduced Grip Strength Domain, the scores were adjusted by sex and BMI, and a score of 1 was given to the lowest 20%. A total summed score for the five domains of 3 or higher indicated frailty, and a score of 1–2 indicated prefrailty. The predictive validity of this tool has been established for negative health outcomes, such as falls, hospital admission, disability, and death [23], and it is the most widely used frailty instrument worldwide [6].

#### 2.5.3. Grip Strength

Grip strength was measured using a dynamometer (JAMAR^®^ hydraulic hand dynamometer; Sammons Preston, Bolingbrook, IL, USA) based on two rounds of measurements for each hand [28]. A previous study that tested the reliability of this tool using the intraclass correlation coefficient (ICC) or reliability coefficient indicated that the values ranged from 0.85 to 0.98 [29]. The test–retest reliability in this study was 0.94.

#### 2.5.4. Short Physical Performance Battery

Physical function was assessed using the SPPB [30], which consists of three domains: a Timed 4 m Walk, Balance, and a Chair Sit-to-Stand Test. The maximum total score, including all three domains, is 12, and a higher score indicates better physical function [30]. The test-retest reliability of this instrument in a previous study was high, at 0.87 (95% CI: 0.07–0.96) [31]. The test-retest reliability in this study was 0.97.

#### 2.5.5. Korean Version of the Community Healthy Activities Model Program for Seniors Questionnaire

Physical activity was measured using the K-CHAMPS. The CHAMPS was originally developed by Stewart and colleagues [32] and was translated, back-translated, modified and adapted for use in South Korea, and validated by Im [33]. This 41-item tool queries about the frequency and duration of participation in an activity in a typical week in the past four weeks. Of the 41 activities, the calorie expenditure/week was calculated for 28 physical activities [33]. The test-retest reliability was 0.96, and Cronbach’s α was 0.64 for all physical activities [33]. Cronbach’s ⍺ was 0.54 in our study. However, Im [33] has stated that it is inappropriate to test the internal consistency of the K-CHAMPS items using the Cronbach’s α, as the instrument is designed to measure various levels of physical activities.

#### 2.5.6. Mini Nutritional Assessment

Nutritional state was measured with the Mini Nutritional Assessment (MNA), originally developed by Rubenstein and colleagues [34] and adapted into Korean and provided free of charge by the Nestle Nutrition Institute. This 18-item tool consists of 6 items for the screening test and 12 items for the main assessment. Each item is weighted according to the response, and the screening test score and main assessment score are summed for a maximum score of 30. The total scores are classified into 0–16 (malnutrition), 17–23 (at risk of malnutrition), and ≥24 (normal). The internal consistency of the scale measured using Cronbach’s α was 0.92 at the time of development and 0.70 in this study.

#### 2.5.7. Geriatric Depression Scale Short Form-Korea Version

Depression was measured using the GDSSF-K, which was originally developed by Yesavage and Sheikh [35] and translated, validated, and standardized in Korean by Ki [36]. This tool consists of 15 items, and a score of 0 or 1 is given for responses of “no” or “yes,” respectively. The positive items 2, 7, 8, 11, and 12 are reverse-scored, and the total score is classified into ≤5 (normal), 6–9 (moderate depression), and ≥10 (major depressive disorder). The scale’s internal consistency measured using Cronbach’s α was 0.88 at the time of development and 0.87 in this study.

#### 2.5.8. ENRICHD Social Support Instrument

Social support was measured using the ESSI. This tool has been widely used in previous studies on community-dwelling older adults such as the Established Populations for Epidemiologic Studies of the Elderly or the Health and Retirement Study, and it was adapted by Jeon and two colleagues [37] with content validity established by a US geriatric health research expert following reverse translation [37,38]. The social support aspect consists of six subscales, including emotional, informational, and instrumental support, and responses of “yes” or “no” are given for each item. “Yes” is given a score of 1, and the scores are summed to generate the total score. A higher total score indicates better social support [39]. The scale’s internal consistency measured using Cronbach’s α was 0.93 at the time of development and 0.73 in this study.

#### 2.5.9. Goal Attainment Scale

The GAS was developed by Kiresuk and Sherman [40] to measure mental health treatment outcomes, and it has been widely used in several other fields, such as education, social welfare, and nursing [25]. It is based on interactions between the examiner and participant. It was useful for assessing the level of goal achievement for each participant in our study as it assisted with appropriate goal selection by helping to identify feasible goals at the time of goal setting and enabling measurement of the level of achievement or effects of the intervention according to individual goals [25,41]. In general, a 5-point scale consisting of −2, −1, 0, +1, and +2 is widely used, with 0 indicating the anticipated outcome or attainable level of a goal [25]. If the anticipated outcomes are not met, a score of −1 or −2 is given depending on the magnitude of the discrepancy, and +1 and +2 indicate that the participant surpassed the target level of the goal or attained optimal results [25].

### 2.6. Data Collection

The data were collected using self-administered or face-to-face interviews at three times: screening test, pretest, and posttest at a senior center in Ilsandong district, Goyang city, in the order of screening test, baseline survey, intervention (12 weeks of PNIF), post-intervention survey, and four sessions of frailty prevention education for the control group. The study lasted for approximately four months from 2 March 2017 to 30 June 2017, and all processes were conducted within the senior center. We developed a clear standard and protocol for the questionnaire survey and anthropometric measurements to minimize variation across 3–6 data collectors, and data collectors reached an agreement on the assessment results to ensure the reliability of the data collection process.

### 2.7. Statistical Analysis

The collected data were analyzed using SPSS version 21 (IBM Corp., Armonk, NY, USA). Participants’ demographic characteristics were analyzed with descriptive statistics, and the normality of each parameter was tested with the Shapiro–Wilk test. The baseline homogeneity between the intervention and control groups was tested using the chi-square test, Fisher’s exact test, the independent *t*-test, and the Mann–Whitney *U* test. Normally distributed dependent variables (left-hand and right-hand grip strength, GDSSF-K) were analyzed with the independent *t*-test, while the SPPB scores, K-CHAMPS scores, MNA scores, ESSI scores, and CHS frailty index were analyzed using the Mann–Whitney *U* test. Differences between the intervention and control groups before and immediately after the intervention were analyzed using the chi-square test, Fisher’s exact test, the independent *t*-test, and the Mann–Whitney *U* test.

## 3. Results

### 3.1. Demographic, Clinical Characteristics and Outcome Variables at Baseline

Table 1 shows the baseline demographic, clinical characteristics, and outcome variables of the participants. There were no statistically significant differences in any of the baseline parameters between the intervention and control groups.

### 3.2. Post-Intervention Outcome Comparison

#### 3.2.1. Cardiovascular Health Study Frailty Index

After the 24-session PNIF over 12 weeks, the CHS frailty index decreased from 1.45 to 0.70 in the intervention group but increased from 1.25 to 1.80 in the control group, indicating a significant difference between the two groups (*p* < 0.001; Table 2). Furthermore, none of the participants in the intervention group were categorized as being in the frailty stage, while five participants (25%) in the control group progressed to the frailty stage, indicating that the severity of their frailty worsened.

#### 3.2.2. Grip Strength

There were significant changes in left-hand (*p* = 0.022) and right-hand (*p* = 0.009) grip strength in both groups (Table 2). In particular, left-hand grip strength increased by 2.65 kg (*SD =* 4.63) in the intervention group but decreased by 1.35 kg in the control group. Right-hand grip strength increased by 5.05 kg in the intervention group but increased by 0.75 kg in the control group.

#### 3.2.3. Short Physical Performance Battery

There were significant differences in the change of the SPPB scores between the two groups (*p* = 0.007; Table 2). The intervention group’s average SPPB score increased from 10.30 to 10.90 (*SD =* 1.52), while the control group’s score decreased slightly from 9.70 to 9.10 (*SD =* 1.94).

#### 3.2.4. Korean Version of the Community Healthy Activities Model Program for Seniors Questionnaire

There were also significant differences in the change of the K-CHAMPS scores (*p* = 0.009; Table 2). The mean K-CHAMPS score for the intervention group increased from 2582.53 to 3471.19 (*SD =* 1990.50), while the control group’s mean score decreased from 1631.43 to 1457.44 (*SD =* 1393.20).

#### 3.2.5. Mini Nutritional Assessment

The change in the MNA score was analyzed using the Mann–Whitney *U* test, and significant differences were observed (*p* = 0.018; Table 2). While the MNA score increased after the completion of the program in both the intervention group and the control group by 2.58 (*SD =* 2.42) and 0.57 (*SD =* 2.07), respectively, there was a marked difference in the degree of increase between the two groups. Furthermore, where none of the participants in the intervention group were malnourished, with a score of below 17 at the post-intervention survey, two participants in the control group were malnourished.

#### 3.2.6. Geriatric Depression Scale Short Form-Korea Version

Changes in the GDSSF-K score were analyzed using the independent *t*-test, and there were statistically significant differences between the two groups (*p* = 0.001; Table 2). Prior to the intervention, GDSSF-K scores were higher in the intervention group (*M =* 6.15, *SD* = 3.80) than in the control group (*M =* 4.35, *SD* = 3.39). However, after the program, the intervention group’s score was 0.9 lower than that of the control group.

#### 3.2.7. ENRICHD Social Support Instrument

After the intervention, there were no statistically significant between-group differences in ESSI scores (*p* = 0.779; Table 2).

### 3.3. Goal Attainment Scale Analysis Results

**Figure 4:**
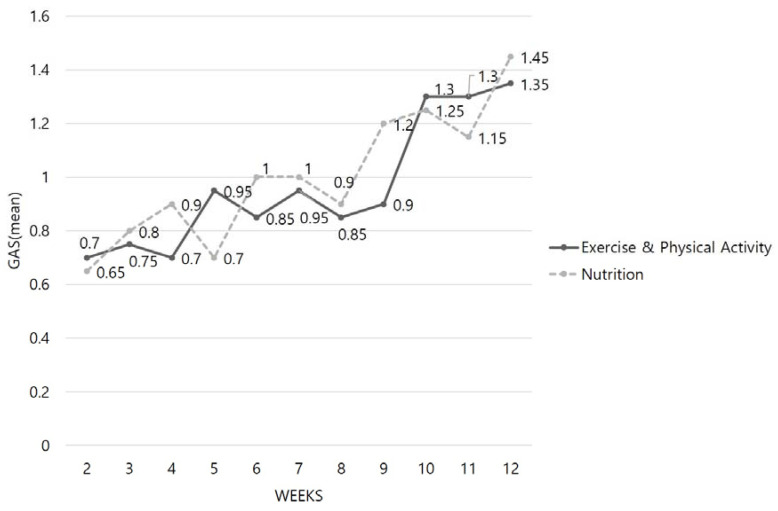
Changes in the average goal attainment scale in the intervention group over 12 weeks.

Figure 4 shows the weekly average Goal Attainment Scale scores for the intervention group. While the average Goal Attainment Scale score was between 0 to <1 until midway through the program, it gradually increased in the later stages of the program, reaching a value of one or higher for the physical activity intervention and nutrition intervention by Week 10. Subsequently, the Goal Attainment Scale score continued to increase slightly.

## 4. Discussion

This study aimed to evaluate the effects of a PNIF on frailty prevention, grip strength, physical function, physical activity, nutrition, depression, and social support among community-dwelling older adults. There was a significant difference in the CHS Frailty Index, a major dependent variable of this study. The positive outcomes in grip strength, physical function, physical activity, nutrition, and depression parameters and the use of an individualized, person-centered strategy seem to have contributed to improving frailty, a multidimensional construct. These results are similar to the significant reduction of frailty reported by Vries and colleagues [42], who assessed the effectiveness of a person-centered intervention.

Our results revealed that the participants’ grip strength significantly increased in both hands compared to those who did not participate in the program. This finding is consistent with the results of an Australian study that conducted a multifactorial intervention and observed significant changes in grip strength after 12 weeks of an intervention [12], and those of a Korean study that provided a health promotion program for frail older adults and also observed significantly increased left-hand and right-hand grip strength in the intervention group [15]. These results are believed to be due to regular upper limb strengthening exercises through group exercises and individual exercise goal selection. During the once per week exercise and physical activity interventions, patients repeatedly performed upper body strengthening exercises using two 0.5-kg dumbbells under the guidance of a nurse. Patients were also encouraged to perform upper body exercise at home using dumbbells or 500-mL water bottles according to the goals they had set in consideration of their capacities. Most older adults were well aware of the importance of aerobic exercises, such as walking, but were unaware of the importance of muscle training [43]. As reduced muscle mass and strength are the major factors contributing to frailty, there is an urgent need for efforts to develop exercise programs that promote the importance of moderate-intensity muscle training and motivate people to participate in these exercises.

In addition to grip strength, the SPPB scores also increased significantly. A previous study that analyzed the effects of an exercise program for community-dwelling prefrail older adults also reported a significant difference in the mean SPPB score between the intervention and control groups after 12 weeks, consistent with our results [44]. According to a recent study, exercise interventions for older adults who are frail should consist of progressive moderate-intensity exercise for at least three 30–45-min sessions per week in order to be effective [45]. Although the exercise intervention in our program was only a single-session intervention, the results we observed are speculated to be attributable to the fact that participants set and achieved their own goals to engage in moderate-intensity muscle training even at home. As studies have reported that a low SPPB score not only increases the incidence of disability and admission to hospitals and long-term care facilities but also has an adverse impact on the mortality rate [46], the improvement in the SPPB score observed in our study is a notable indicator that the negative health outcomes of frailty were decreased. As a strategy to delay further muscle atrophy and weakening due to aging, older adults should be instructed to regularly engage in moderate-intensity muscle training to increase their independence and quality of life in older adulthood.

After the 12-week intervention, K-CHAMPS scores decreased in the control group, but increased among the intervention group, a significant between-group difference. This result could be linked to the psychosocial intervention in our program. Previous studies that examined predictors of physical activity in home-dwelling older adults reported that physical activity participation among older adults was strongly associated with psychological factors [47,48]. Park and Park [47] emphasized that older adults with high levels of depressive symptoms or stress engage in fewer physical activities and stated that physical activity-promoting programs for older people should be designed as person-centered programs that encompass both physical health problems caused by aging and various psychosocial factors. Our program encompassed a psychosocial intervention, which is speculated to have led to a more positive impact on increasing physical activity, but which may be readily neglected by frailty prevention programs. Furthermore, providing opportunities to participate in this program conducted at the senior center probably also contributed to increasing physical activity. Some participants mentioned that having to visit the senior center twice per week for this program increased their interactions with other program participants, increased their interest in other programs hosted at the senior center, and improved their confidence. A survey in 2014 regarding how older adults spent their leisure time indicated that “resting” was the most common activity (90.2%), of which “watching TV” accounted for the majority (82.4%) [2]. Expanding the infrastructure for senior centers and consequently increasing participation in senior leisure programs would also lead to increased physical activity, which is associated with various health benefits. Thus, the implementation of applicable senior welfare policies is urgently needed.

There was also a significant between-group difference in MNA scores. We believe that this result was caused by providing an opportunity for participants to correct their dietary patterns through group nutrition education, individual goal setting, and monitoring. Dietary improvements also require nurses’ continued attention and support. Older adults are at a higher risk for malnourishment than other age groups and have a higher prevalence of chronic diseases, such as hypertension and diabetes mellitus, which are closely associated with diet. Therefore, despite the crucial importance of dietary management in older adulthood, nutrition-related projects in seniors still have not been launched [49]. A Korean study by Lee and colleagues [49] analyzed the needs for nutritional and diet management programs and reported that while 46.9% of senior center users claimed to need nutritional education and counseling, only 19.8% of them had actually received diet education in the past. In addition, they reported that the greatest problem with Korean nutrition-related programs for seniors was the shortage of personnel for nutrition and diet education [49]. As nutritional management is as important for frailty prevention as regular exercise and physical activity, it is crucial for the government to ensure additional staffing for nutrition counseling and to provide active support for the improvement of diet in older adults.

The post-intervention survey revealed a significant difference in depression (GDSSF-K) scores between the two groups. A Korean study that examined changes in depression after implementing a health promotion program for older adults who are frail also reported that the program participants had significantly lower levels of depressive symptoms than the non-participants [15]. A previous study examining the associations among frailty, depression, and anxiety among older adults reported that those who are prefrail and frail experienced more anxiety and depressive symptoms than their healthier counterparts [50]. Mental health, which can be affected by conditions such as depression and stress, is as important as physical health and should not be overlooked for frailty prevention.

Nevertheless, there was no significant between-group difference in ESSI scores. Multiple studies have documented that the prevalence of prefrailty and frailty in older adulthood significantly increases in association with decreased social networking and social support, indicating that social support is a major predictor of frailty comparable to psychological factors such as depression and anxiety [37,51]. However, when developing programs to boost social support, individuals’ tendencies, areas of residence, and cultural traits must be considered. The ESSI, which was used in this study, includes items such as “Do you have somebody to ask for help with small things or household chores when needed?” and “Do you have someone who wholeheartedly helps you when you must make an important decision or have hardships?” Most older adults thought of their “family” when responding to these questions and tended to consider asking for help from non-family members such as neighbors or friends as bothersome for them. Moreover, they lacked confidence in developing new interpersonal relationships deep enough to be able to depend on other people at the senior center. Such tendencies must be taken into consideration when developing social support programs, and longer (≥12 weeks) interventions and special attention may be needed to facilitate the development of relationships that are sufficient to elevate social support.

The intervention strategy used in this study was based on the person-centered processes of the person-centered nursing framework developed by McCormack and McCance [52]. The strategy pertinent to shared decision-making and empathy seems to have served as a useful strategy for “goal setting and monitoring,” which is considered a primary component of person-centered care (PCC). A systematic review analyzing the effects of PCC concluded that PCC is an effective intervention strategy given that the major dependent variable improved significantly in eight out of 11 studies [53]. Our program was designed so that participants were able to set their own goals for exercise and nutrition in order to respect their right to self-determination and their individual preferences and abilities as much as possible. This process is believed to have had a positive impact on attaining the ultimate goal—avoiding frailty. Lecture-based group education has inherent restrictions in dealing with the diverse problems and complex needs of each older adult. In particular, because frailty among older adults occurs as a result of complex interactions between aging and physical and psychosocial factors, and because individuals have distinct needs, a person-centered strategy is essential. For implementation of PNIF, considerable effort, time, and nursing staff are required at senior centers. In fact, healthcare managers in the US pinpointed insufficient financial resources and a lack of staffing for the workload as barriers to person-centered care [20]. Thus, it cannot be guaranteed that beneficial health-promotion programs can be implemented even if developed. Expanding healthcare professional staffing at senior centers should be prioritized to resolve the major health problems among the growing aging population and implement practically needed health-promotion programs. Such changes would improve older adults’ health and quality of life and contribute to decreasing national healthcare expenditures.

This study had several limitations. First, it was conducted at one senior center in Gyeonggi Province, so subsequent studies should verify the effectiveness of this program in other regions. Second, the effectiveness of the program was verified based only on a comparison of the baseline and post-intervention surveys after a 12-week intervention. Longer-term follow-ups, such as six months or one year, are needed. Third, mixed-methods studies, including qualitative research, should be conducted to obtain an in-depth understanding and assessment of our findings.

## 5. Conclusions

Our multifactorial person-centered nursing intervention for frailty among community-dwelling older adults who are prefrail improved grip strength, physical function, physical activity, nutritional status, and reduced depressive symptoms. Therefore, this program can be considered effective in preventing frailty. Verifying the effectiveness of this program would contribute to preventing frailty, maintaining functional independence among older people, and improving their quality of life. It would also benefit South Korea as a whole by mitigating a variety of social problems related to population aging. Replicating this research among participants in various regions and from other cultures is needed to generalize these results. More long-term follow-up studies and additional statistical analyses are needed to confirm the persistence of the effects of the intervention program.

## Figures and Tables

**Table 1:** Homogeneity test of demographic, clinical characteristics, and outcome variables.

Characteristics		Intervention Group (*n* = 20)	Control Group (*n* = 20)	χ^2^ or *t*	*p*
		*n* (%) or Mean (SD)	*n* (%) or Mean (SD)		
Age (year)		77.10 (6.40)	79.30 (4.89)	−1.23	0.227
Sex	Male	4 (20.0%)	8 (40.0%)	1.91	0.168
	Female	16 (80.0%)	12 (60.0%)		
Educational level	None	0 (0.0%)	2 (10.0%)	4.65 *	0.320
	Elementary school	5 (25.0%)	8 (40.0%)		
	Middle school	4 (20.0%)	5 (25.0%)		
	High school	7 (35.0%)	3 (15.0%)		
	University or above	4 (20.0%)	2 (10.0%)		
	Mean (SD)	10.10 (4.10)	7.45 (4.72)	1.90	0.066
Marital status	Married	8 (40.0%)	7 (35.0%)	0.28 *	1.000
	Widowed	10 (50.0%)	11 (55.0%)		
	Divorced	0 (0.0%)	0 (0.0%)		
	Separated	2 (10.0%)	2 (10.0%)		
	Not married	0 (0.0%)	0 (0.0%)		
Family type	Solitary	10 (50.0%)	6 (30.0%)	2.60	0.295
	Spouse	7 (35.0%)	7 (35.0%)		
	Others	3 (15.0%)	7 (35.0%)		
Religion	Yes	19 (95.0%)	18 (90.0%)	7.21 *	0.050
	No	1 (5.0%)	2 (10.0%)		
Perceived economic status	Upper	0 (0.0%)	0 (0.0%)	3.60	0.058
	Middle	13 (65.0%)	7 (35.0%)		
	Lower	7 (35.0%)	13 (65.0%)		
Number of chronic diseases		4.20 (2.33)	3.80 (2.40)	−0.52 ^a^	0.620
MMSE-KC		26.25 (0.68)	25.75 (0.52)	0.59 ^b^	0.562
CHS frailty index		1.45 (0.51)	1.25 (0.44)	−1.31 ^a^	0.289
Grip strength	Left	22.40 (7.16)	21.05 (7.19)	0.60 ^b^	0.556
	Right	21.70 (7.84)	21.10 (7.25)	0.25 ^b^	0.803
SPPB		10.30 (1.49)	9.70 (1.81)	−1.07 ^a^	0.301
K-CHAMPS (Kcal/week)		2582.53 (1569.18)	1631.43 (1360.46)	−1.84 ^a^	0.068
MNA		22.50 (3.48)	21.68 (4.26)	−0.91 ^a^	0.369
GDSSF-K		6.15 (3.80)	4.35 (3.39)	1.58 ^b^	0.122
ESSI		5.25 (1.12)	5.00 (1.34)	−0.52 ^a^	0.640

Notes: * Fisher’s exact test, ^a^ Mann–Whitney *U* Test, ^b^ Independent t-test. Abbreviations: SD, standard deviation; MMSE-KC, Mini Mental State Examination in the Korean version of the Consortium to Establish a Registry for Alzheimer’s Disease assessment packet; CHS, Cardiovascular Health Study; SPPB, Short Physical Performance Battery; K-CHAMPS, Korean version of the Community Healthy Activities Model Program for Seniors Questionnaire; MNA, Mini Nutritional Assessment; GDSSF-K, Geriatric Depression Scale Short Form-Korea Version; ESSI, ENRICHD Social Support Instrument.

**Table 2:** Comparison of variables between the intervention and control group.

Characteristics	Group	Pretest	Posttest	Difference	*t*/Z	*p*
		Mean (SD)	Mean (SD)	Mean (SD)		
CHS frailty index	Intervention group (*n* = 20)	1.45 (0.51)	0.70 (0.73)	−0.75 (0.85)	−3.558 ^a^	<0.001
	Control group (*n* = 20)	1.25 (0.44)	1.80 (1.01)	0.55 (1.10)		
Grip strength, Left-hand (kg)	Intervention group	22.40 (7.16)	25.05 (5.07)	2.65 (4.63)	2.387 ^b^	0.022
	Control group	21.05 (7.19)	19.70 (7.36)	−1.35 (5.90)		
Grip strength, Right-hand (kg)	Intervention group	21.70 (7.84)	26.75 (4.72)	5.05 (5.72)	2.733 ^b^	0.009
	Control group	21.10 (7.25)	21.85 (6.69)	0.75 (4.10)		
SPPB	Intervention group	10.30 (1.49)	10.90 (1.52)	0.60 (1.27)	−2.741 ^a^	0.007
	Control group	9.70 (1.81)	9.10 (1.94)	−0.60 (1.35)		
K-CHAMPS	Intervention group	2582.53 (1569.18)	3471.19 (1990.50)	888.65 (1858.09)	−2.570 ^a^	0.009
	Control group	1631.43 (1360.46)	1457.44 (1393.20)	−174.00 (810.74)		
MNA	Intervention group	22.50 (3.48)	25.08 (2.44)	2.58 (2.42)	−2.361 ^a^	0.018
	Control group	21.68 (4.26)	22.25 (4.54)	0.57 (2.07)		
GDSSF-K	Intervention group	6.15 (3.80)	4.75 (3.80)	−1.40 (2.30)	−3.750 ^b^	0.001
	Control group	4.35 (3.39)	5.65 (4.18)	1.30 (2.25)		
ESSI	Intervention group	5.25 (1.12)	5.10 (1.29)	−0.15 (1.42)	−0.322 ^a^	0.779
	Control group	5.00 (1.34)	4.60 (1.70)	−0.40 (1.88)		

Notes: ^a^ Mann–Whitney *U* Test, ^b^ Independent *t*-test. Abbreviations: SD, standard deviation; MMSE-KC, Mini Mental State Examination in the Korean version of the Consortium to Establish a Registry for Alzheimer’s Disease assessment packet; CHS, Cardiovascular Health Study; SPPB, Short Physical Performance Battery; K-CHAMPS, Korean version of the Community Healthy Activities Model Program for Seniors Questionnaire; MNA, Mini Nutritional Assessment; GDSSF-K, Geriatric Depression Scale Short Form-Korea Version; ESSI, ENRICHD Social Support Instrument.

## Appendix A

**Table A1:** Topics of the nutritional and psychosocial support intervention sessions in a person-centered nursing intervention for frailty.

Week	Nutritional Intervention Session	Psychosocial Support Intervention
1	Nutritional characteristics of older adults Importance of nutritional management for older adults	Understanding and introducing oneself
2	Causes of older adults’ malnutrition and understanding problems with one’s eating habits Setting individual goals	Definition of mental health Causes and symptoms of stress in older adults
3	Dietary guidelines for older adults Last week’s goal evaluation and setting individual goals	Depression among older adults Telling the story about one’s daily life in three words
4	The six basic food groups (food composition bicycles) Last week’s goal evaluation and setting individual goals	Drawing one’s current mood on a sketchbook and having a conversation about it
5	Group advice about the drawing of one’s dinner menu Last week’s goal evaluation and setting individual goals	Making a face of someone whom one is grateful for with toy clay, part 1
6	Five nutrients that can easily be deficient among older adults Last week’s goal evaluation and setting individual goals	Making a face of someone whom one is grateful for with toy clay, part 2
7	Intermediate quiz session Last week’s goal evaluation and setting individual goals	Dancing to a cheerful song Massaging each other
8	Simple recipes for older adults, part 1 Last week’s goal evaluation and setting individual goals	Introducing happy memories about one’s childhood after drawing them in a sketchbook
9	Simple recipes for older adults, part 2 Last week’s goal evaluation and setting individual goals	Telling about one’s past or a present dream
10	Simple recipes for older adults, part 3 Last week’s goal evaluation and setting individual goals	Meditation session
11	Sharing examples of applying simple recipes Introducing one’s own helpful and simple recipe Last week’s goal evaluation and setting individual goals	“I praise you” session
12	Sharing my changes in dietary habits Praising each other for their positive changes (completion ceremony)	Making a certificate of merit for oneself

## Appendix B

**Table A2:** Outcome measurements of this study.

	Outcome Variables	Data Collection Method	Test-Retest Reliability or Cronbach’s Alpha
1	CHS Frailty Index	Self-administered or face-to-face interview Physical measurement	
2	Grip strength (by JAMAR^®^ hydraulic hand dynamometer)	Physical measurement	0.94
3	SPPB	Physical measurement	0.97
4	K-CHAMPS	Self-administered or face-to-face interview	0.54
5	MNA	Self-administered or face-to-face interview	0.70
6	GDSSF-K	Self-administered or face-to-face interview	0.87
7	ESSI	Self-administered or face-to-face interview	0.73
8	GAS	Self-administered or face-to-face interview	

Abbreviations: CHS, Cardiovascular Health Study; SPPB, Short Physical Performance Battery; K-CHAMPS, Korean version of the Community Healthy Activities Model Program for Seniors Questionnaire; MNA, Mini Nutritional Assessment; GDSSF-K, Geriatric Depression Scale Short Form-Korea Version; ESSI, ENRICHD Social Support Instrument; GAS, Goal Attainment Scale.
